# Integrated Transcriptome and Metabolome Analysis of Mature Stage Sand Pear Fruit Response to High-Temperature Stress

**DOI:** 10.3390/plants14172776

**Published:** 2025-09-04

**Authors:** Yu-Xuan Li, Jia-Bei Cai, Xiao Liu

**Affiliations:** College of Horticulture and Landscape Architecture, Yangzhou University, Yangzhou 225009, China

**Keywords:** sand pear, high temperature, watercore, transcriptome, metabolomic

## Abstract

Sand pear is a fruit tree crop with high economic value, widely cultivated in East Asia. However, ripening fruits often suffer from high-temperature stress, which has adverse effects on the quality and yield of the fruit. In this study, we perform high-temperature treatment on mature stage ‘Housui’ pear fruits. The results showed that heat stress decreased fruit firmness and mineral elements, as well as lead to the flesh appearance of watercore. High temperature induces H_2_O_2_, MDA, and the antioxidant enzyme activity including SOD, APX, POD, and CAT were significantly increased. Transcriptome and metabolomic analyses revealed that heat stress up-regulated genes related to sucrose synthesis (SPS) while down-regulating those involved in sucrose degradation (SS and NI), resulting in sucrose accumulation. Moreover, the expression of sorbitol dehydrogenase (SDH) and sorbitol transporter (SOT) genes was markedly suppressed, leading to sorbitol accumulation and impaired transport, which promoted watercore development. High temperature also stimulated the expression of ethylene synthesis genes, accelerating abnormal ripening of fruits. In addition, high temperature decreased the accumulation of organic acid and bioactive compounds. Additionally, several antioxidant enzymes genes, five heat shock transcription factors (HSFs) and 34 heat shock protein (HSP) genes were significantly up-regulated. Together, these findings provided new insights into the transcriptional response and metabolomic reprogramming of sand pear response to high-temperature stress.

## 1. Introduction

High-temperature stress has emerged as a significant limiting factor for stable crop production globally. Under high-temperature conditions, plant transpiration increases, leading to rapid water loss. Roots may compensate for the increased water loss caused by transpiration, but this compensation is often insufficient to protect the fruit, which can suffer from dehydration, impaired development, or heat-induced damage [1]. Moreover, high temperatures can disrupt plant photosynthesis by reducing the activity of photosynthetic enzymes, damaging chloroplast structures, and decreasing the synthesis of photosynthate [2]. Additionally, increased respiration diverts fixed carbon away from biosynthetic pathways and towards energy production through increased TCA cycle activity. This results in a net loss of assimilated carbon as CO_2_, reducing the overall carbon availability. The primary consequences are a shortage of substrates for the synthesis of structural and storage compounds, which directly translates into diminished biomass accumulation, reduced fruit yield, lower seed weight, and impaired quality of harvestable organs [3]. In response to high-temperature stress, plants initiate a series of physiological and molecular responses. Physiologically, plants lower their cell osmotic potential and retain cellular moisture by increasing the production of osmoregulatory substances such as proline and betaine [4]. At the same time, antioxidant enzyme systems, including superoxide dismutase (SOD), peroxidase (POD), catalase (CAT), and ascorbate peroxidase (APX), exhibit heightened activity, clearing excess reactive oxygen species (ROS) induced by high temperatures and mitigating oxidative damage [5]. At the molecular level, heat shock transcription factors (HSFs) are activated to regulate the expression of heat shock protein genes, leading to the synthesis of heat shock proteins (HSPs) that assist in the proper folding and repair of plant proteins, maintaining intracellular protein homeostasis and enhancing plant tolerance to high temperatures [6].

High temperatures can directly or indirectly lead to various physiological disorders in fruit crops. The most prevalent issue is that high temperatures, alone or in conjunction with intense sunlight, result in sunburn on the fruit’s skin [7]. Elevated temperatures can also cause other problems, such as fruit cracking, fruit rust, and cork disorder. For instance, tomato fruit cracking rates have been observed to increase when the ambient temperature surpasses 30 °C, and the temperature difference between the day’s high and low exceeds 20 °C [8]. ‘Hongro’ apples exhibited a higher incidence of fruit cracking and spot disorders in the heat treatment group compared to those in the water-irrigated and control groups [9]. Furthermore, watercore is a frequent physiological disorder in Rosaceae crops, including pears and apples. Current research indicates a strong correlation between pre-harvest high-temperature stress and the incidence of watercore [10,11], yet the molecular mechanisms behind high-temperature-induced watercore formation require further investigation.

The sand pear is one of China’s significant fruit crops, known for its high intrinsic quality and economic value, and is cherished by consumers. The cultivation of sand pears is primarily concentrated in the middle and lower reaches of the Yangtze River, with the fruit typically maturing in summer. Despite their relative tolerance to high temperatures, recent years have seen the frequent and continuous occurrence of extreme summer temperatures due to global climate change. These extreme conditions have significantly and adversely affected the stable production of sand pears. Currently, research into the effects of high temperatures on sand pears primarily focuses on seedlings or trees and is largely conducted at the physiological level. There remains limited research concerning the response of mature fruits to high-temperature stress. Multiple omics technologies, such as transcriptomics and metabolomics, comprehensively analyze the molecular mechanisms of plant response to abiotic stresses such as drought and heat from a systems biology perspective [12,13]. The transcriptome reveals the expression regulatory network of key genes, while the metabolome directly reflects the physiological and biochemical changes in the plant under stress. Integrating multiple omics data can accurately mine core stress response genes and metabolites, clarify their interactions, and construct a complete regulatory pathway from genes to phenotypes. In the current study, we employed a multi-omics approach to elucidate the changes in pathways related to antioxidant enzyme synthesis, metabolism of intrinsic quality components such as sugars and acids, hormone biosynthesis, as well as heat shock proteins and heat shock transcription factors under high-temperature stress. The findings aim to provide a theoretical foundation for further investigations into its preservation mechanisms.

## 2. Results

### 2.1. The Effect of High-Temperature Treatment on the Temperature, Firmness, and Intrinsic Phenotype of Pear Fruits

**Figure 1:**
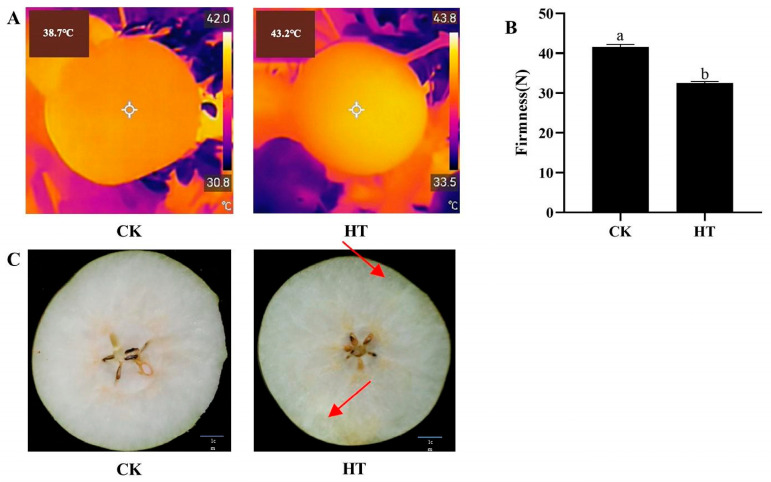
Effects of high-temperature treatment on fruit temperature (**A**), firmness (**B**), and intrinsic phenotype (**C**). The red arrow represents the watercore area. Each column represents a mean ± standard error (*n* = 3). Significance was determined by one-way ANOVA with Tukey’s test and represented in lowercase letters (*p* < 0.05).

As illustrated in Figure 1A, the surface temperature of the fruit increased significantly following high-temperature treatment. After long-term high-temperature treatment, the firmness of the fruit significantly decreased by about 22% (Figure 1B). In recent years, it has been found that high temperatures can also cause watercore in sand pears during production. There were also symptoms of watercore inside the high-temperature-treated fruit (Figure 1C).

### 2.2. The Effect of High Temperature on the MDA, H_2_O_2_, and Activity of Antioxidant Enzymes in Fruits

**Figure 2:**
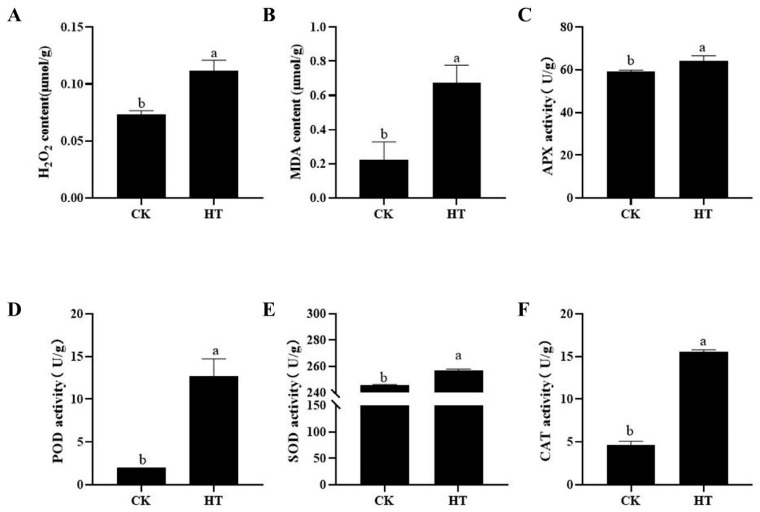
The effect of high temperature on the MDA (**A**), H2O2 (**B**), and enzyme activity of APX (**C**), POD (**D**), SOD (**E**), and CAT (**F**) in fruits. Each column represents a mean ± standard error (*n* = 3). Significance is determined by one-way ANOVA with Tukey’s test and represented in lowercase letters (*p* < 0.05).

In this study, high temperature caused a significant accumulation of MDA and H_2_O_2_ content in the fruit pulp (Figure 2A,B). In addition, the activities of various antioxidant enzymes, including POD, SOD, CAT, and APX were significantly increased by high temperature induction (Figure 2C–F). Especially, the activity of POD and CAT significantly increased by 5.3 and 2.4 times, respectively.

### 2.3. The Effect of High Temperature on the Content of Mineral Elements in Fruits

**Figure 3:**
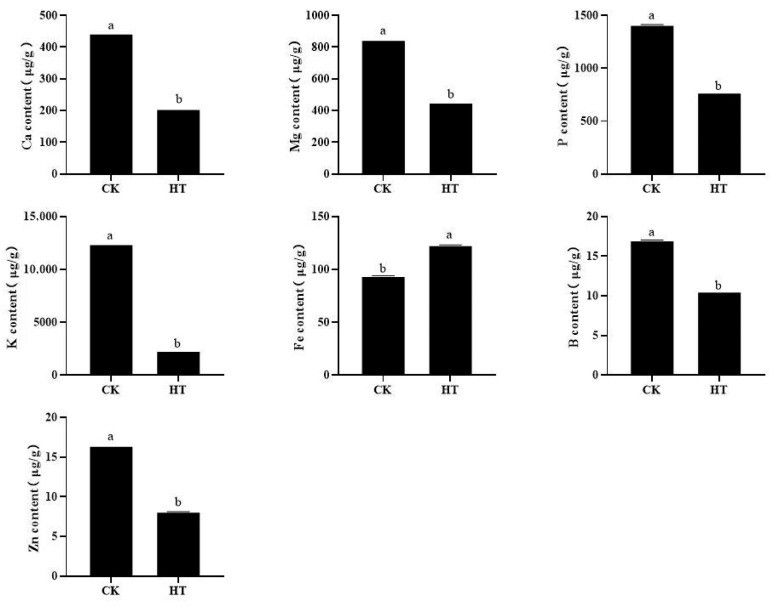
The effect of high temperature on the mineral elements in fruit pulp. Each column represents a mean ± standard error (*n* = 3). Significance is determined by one-way ANOVA with Tukey’s test and represented in lowercase letters (*p* < 0.05).

As shown in Figure 3, the contents of Ca, Mg, P, K, B, and Zn all significantly decreased, by 53.68%, 46.70%, 45.34%, 81.31%, 37.88%, and 50.57%, respectively. Only Fe content significantly increased by 31.00% after high-temperature stress.

### 2.4. Analysis of Differential Metabolites in Fruits Under High-Temperature Stress

**Figure 4:**
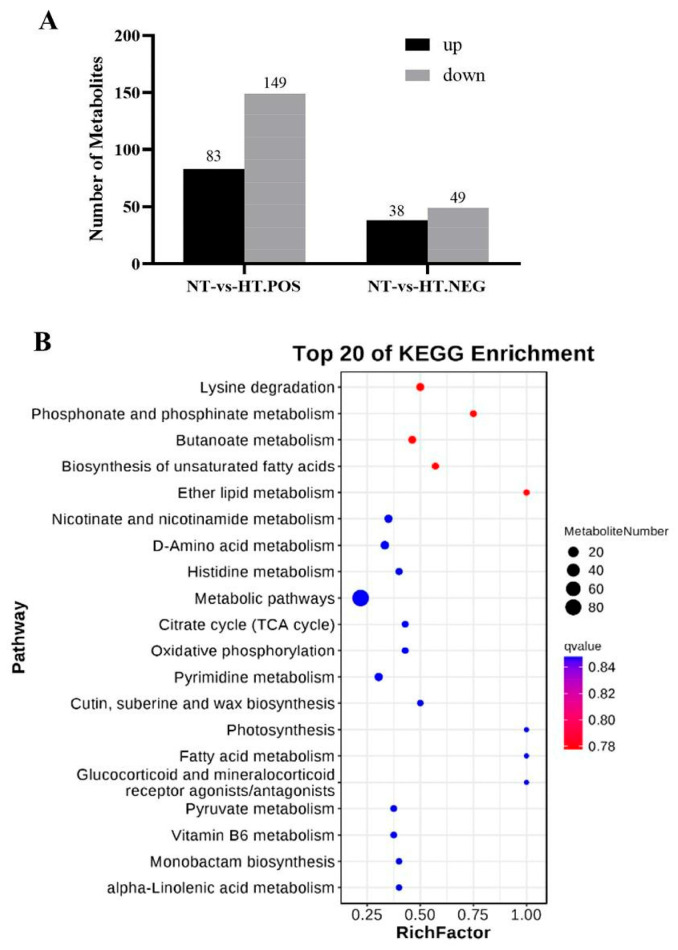
Differential metabolite statistics (**A**) and KEGG analysis (**B**). NT and HT refer to normal temperature and high temperature, respectively.

Metabolome analysis was conducted on the fruit pulp after high-temperature treatment. As shown in Figure 4A, there were a total of 232 differential metabolites in the positive ion mode, with 83 increased and 149 decreased, respectively; there were a total of 87 differential metabolites in negative ion mode, with 38 increased and 49 decreased, respectively. KEGG analysis shows that differential metabolites were mainly distributed in metabolic pathways such as metabolic biosynthesis of secondary metabolites and amino acid metabolism (Figure 4B).

### 2.5. Changes in Main Sugar and Acid Components in Fruits Under High-Temperature Stress

|Log_2_^FC^| > 0 and variable importance in projection (VIP) ≥ 1 were considered differential metabolites. As shown in Table 1, after high-temperature stress, the content of main sugar metabolites such as fructose, sucrose, and sorbitol in the fruit was significantly increased. In addition, except for a significant increase in succinic acid, the content of malic acid, shikimic acid, quinic acid, and fumaric acid was significantly decreased.

### 2.6. Changes in Bioactive Substances in Fruits Under High-Temperature Stress

Pear fruits also contain various bioactive substances, including polyphenols and amino acids. In this study, the content of various bioactive substances, including amino acids and other phenols, in pear fruit undergoes significant changes after being subjected to high-temperature stress. In amino acid substances, except for an increase in isoleucine, the content of pipecolic acid, γ-aminobutyric acid, methionine, alanine, asparagine, aspartic acid, serine, and glutamic acid significantly decreased. In addition, quercetin, chlorogenate, and gallic acid also had significant reductions (Table 2).

### 2.7. RNA Sequencing and Analysis of Differentially Expressed Genes

**Figure 5:**
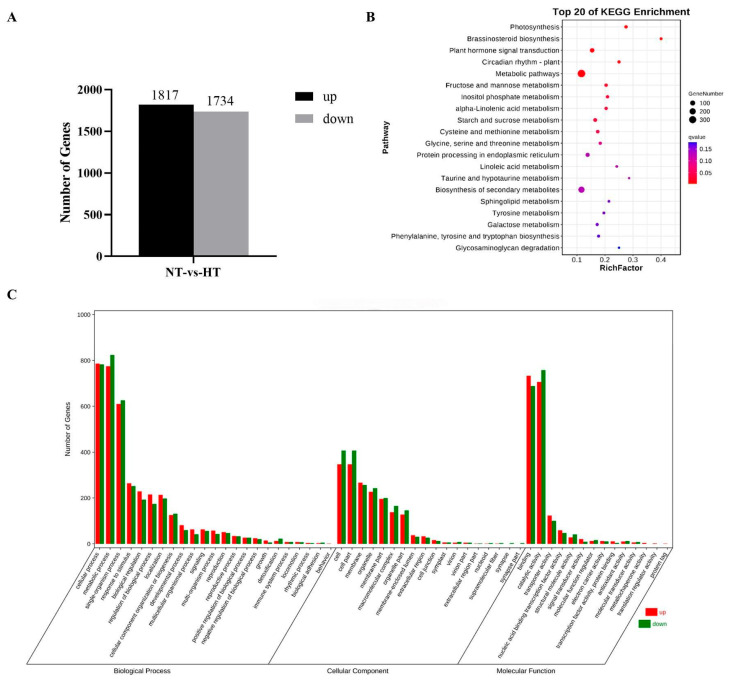
Differential expression gene statistics (**A**), KEGG (**B**), and GO (**C**) analysis. NT and HT refer to normal temperature and high temperature, respectively.

A total of 1817 and 1734 genes were significantly up-regulated and down-regulated in heat-treated pulp, respectively (Figure 5A). All the DEGs were subjected to KEGG and GO analysis. KEGG results showed the DEGs were mainly involved in the pathways of biosynthesis of secondary metabolites, carbohydrate metabolism, plant hormone signal transduction, and amino acid metabolism (Figure 5B). In the GO database, cellular process, metabolic process, and signal-organism process were the most enriched in the ‘biological process’; cell, cell part, membrane, and organelle were the most enriched in the ‘cellular component’ category; and binding and catalytic activity were the most enriched in the ‘molecular function’ category cellular component (Figure 5C). Overall, the genes enriched through metabolic process (1599), cellular process (1569), catalytic activity (1464), binding (1421), and single-organism process (1236) were the most abundant.

### 2.8. Transcriptome Changes in Antioxidant Enzyme Genes in High-Temperature-Treated Fruits

As shown in Table 3, multiple genes related to plant antioxidant enzymes were identified in fruits subjected to high-temperature stress. Among them, the expressions of two POD (LOC103966973, LOC103934252) were significantly reduced, while three POD genes (LOC103964015, LOC103938713, LOC103958862) were significantly increased. One SOD enzyme (LOC103947719) showed a decrease in expression level, while the other enzyme (LOC103956225) showed an increase in expression level. The expression levels of two APX genes (LOC103934327, LOC103948906) and two GST genes (LOC103930887, LOC103958810) were significantly increased.

### 2.9. Transcriptome Changes in Sugar Metabolism-Related Genes in High-Temperature-Treated Fruits

In the process of sucrose metabolism, sucrose phosphate synthase (SPS) is mainly involved in the synthesis of sucrose, while sucrose synthase (SS) and neutral invertase (NI) mainly function in the degradation of sucrose during fruit ripening. Transcriptome analysis revealed an increase in the expression level of one SPS gene (LOC103952486). The expression level of one SS gene (LOC103935319) significantly increased, while the expression levels of two SS genes (LOC103959924, LOC103939687) significantly decreased. The expression levels of two NI genes (LOC103952542, LOC103954772) were significantly reduced, while the expression level of one NI gene (LOC103928220) was significantly increased (Table 4). This also indicates that, under high-temperature conditions, the synthesis of sucrose in the fruit was enhanced while its degradation was reduced, resulting in a significant accumulation of sucrose content in the fruit.

Sorbitol transporter (SOT) and sorbitol dehydrogenase (SDH) are responsible for the transmembrane transport and cytoplasmic degradation of sorbitol, respectively. As shown in Table 4, the expression levels of 10 SDH genes (LOC103933317, LOC103933318, LOC103933319, LOC103960508, LOC103960512, LOC103960507, LOC103960513, LOC103933316, LOC103933312, and LOC103933314) and 4 SOT genes (LOC103929477, LOC103964608, LOC103934620, LOC103936910) were all significantly down-regulated (Table 4).

### 2.10. Differential Genes Related to Ethylene and Abscisic Acid Synthesis

Methionine is activated by ATP and converted to SAM (S-adenosylmethionine) through the action of SAM synthase. SAM is converted to ACC (1-aminocyclopropane-1-carboxylic acid) in the presence of ACC synthase (ACS), and ACC is converted to ethylene with the help of ACC oxidase (ACO). Transcriptome analysis revealed nine differentially expressed ACO genes, of which eight (LOC103946002, LOC103958907, LOC103939367, LOC103943975, LOC103967481, LOC103958927, LOC103958906, LOC103943060) were significantly up-regulated and one (LOC103954990) was significantly down-regulated (Table 5).

ZEP, NCED, and AAO are key enzymes that affect abscisic acid synthesis. Transcriptome analysis revealed that the expression level of one ZEP enzyme gene (LOC103937745) was significantly down-regulated, while the expression level of one NCED enzyme gene (LOC103945979) was significantly down-regulated. The expression level of the key AAO enzyme gene LOC103959275 was significantly up-regulated, while the expression level of LOC103952077 decreased (Table 5). Therefore, long-term high temperatures can inhibit the synthesis of endogenous ABA in pear fruits.

### 2.11. Exploration of Differentially Expressed Heat Shock Proteins and Heat Shock Transcription Factors

As shown in Table 6, 34 HSP genes were screened, and their expression levels were all up-regulated. Among them, eight (LOC103946415, LOC103966141, LOC103964327, LOC103927498, LOC103966169, LOC103953301, LOC103960307, LOC103949874) showed differential expression levels of more than five times. Among the seven HSFs genes, five (LOC103944300, LOC103952243, LOC103955174, LOC103944219, LOC103960090) showed up-regulation of expression levels, while two (LOC103936188, LOC103945943) showed down-regulation of expression levels.

## 3. Discussion

Plants have evolved complex and diverse modes of action or response mechanisms to resist high-temperature stress. At the molecular level, the main regulatory pathways of plants under high-temperature stress include ROS, hormone regulatory pathways, heat stress transcription factor shock proteins, and calmodulin pathways [1]. Long-term high-temperature stress can lead to the accumulation of ROS. Excessive ROS can cause damage to membrane structure, leading to increased membrane permeability and disrupting cellular functions such as ion exchange and water absorption. A large amount of ROS can also lead to protein denaturation and misfolding in the reproductive organs [14]. There are protective enzyme systems in plants that specifically scavenge the excess of ROS accumulation, including SOD, APX, CAT, and POD. Currently, research on high-temperature stress in horticultural crops mostly focuses on seedlings as materials, so high-temperature treatment is relatively easy to operate. However, due to the tall stature of pear trees, it is difficult to accurately fix the temperature of their fruits. Based on previous research, we utilized a constant temperature resistor to create a high-temperature environment around pear fruits. Our research results were consistent with the response of many horticultural crops to high-temperature stress and further demonstrated the effectiveness of high-temperature treatment for fruits.

At present, there are many studies on the impact of high temperature on fruit quality [15,16,17]. High temperatures usually promote abnormal ripening of fruits. Encountering high-temperature stress in the early stages of fruit growth and development usually has adverse effects on the characteristics of fruit enlargement and the accumulation of sweetness in the later stages. However, the changes in fruit quality after being affected by high temperatures during the later stages of fruit enlargement or ripening are different. For example, high temperature changes the metabolism of sorbitol and sucrose in the flesh of ‘ Wonhwang ‘ fruits during the late stage of swelling, and the content of sorbitol and sucrose in the flesh significantly increases during harvest [18]. In this study, we also found a significant increase in the content of major sugar components such as sucrose, fructose, and sorbitol in fruits treated with high temperature. Ethylene, as an important plant hormone, participates in regulating almost all plant growth and development processes, especially playing a key regulatory role in fruit ripening [19]. Research on bananas has found that high temperatures accelerate the decrease in fruit hardness, increase ethylene production, inhibit fruit chlorosis, and lead to abnormal fruit ripening [20]. In plants, the production of ethylene coincides with fruit ripening and plays a key role in coordinating various stages of ripening, including pigment deposition, odor, and texture evolution [19]. Transcriptome analysis revealed that many ACO genes were significantly up-regulated. Therefore, we speculate that high-temperature stress during the ripening period of pear fruit will promote the synthesis of endogenous ethylene, accelerate fruit ripening, and increase fruit sugar content. The increase in ethylene also accelerates the softening of fruits [19], resulting in a significant decrease in pear fruit firmness after high-temperature treatment. In addition, ABA plays an important role in enhancing plant resistance to high temperatures, including stomatal regulation, antioxidant activation, stress signaling, etc. [21]. Some studies have found that high-temperature stress can inhibit the content of endogenous ABA [22]. Long-term high temperatures can inhibit the synthesis of endogenous ABA in pear fruits. Moreover, the long-term high-temperature stress had an inhibitory effect on the content of bioactive substances, affecting fruit quality. These substances play an important role in plant cell antioxidant, ROS scavenging, antibacterial, and antiviral processes, and they also have good effects on human dietary health [23,24].

Watercore is a physiological disorder that is more common in pears and apples, with fruit showing symptoms of waterlogging. Once a pear suffers from watercore, its taste and storage capacity will be greatly reduced [25]. Pear watercore is more common in sand pear varieties with higher intrinsic fruit quality, such as ‘Housui’, ‘Sucui No.1′, ‘Akizuki’, and other main cultivars. High-temperature stress can significantly disrupt the ion homeostasis of plants, which is the balance of ions such as K^+^ and Na^+^ inside and outside the plant cells. This disruption can lead to inhibited plant growth and development [26]. High temperatures have been shown to disturb the uptake, transport, and partitioning of essential mineral elements in strawberries [27]. Pimenta et al. found that warmer temperature affect mineral transport from source to sink tissues in tomato [28]. Previous studies have shown that deficiencies in Ca and B are important causes of watercore [29]. Because Ca^2+^ plays a crucial role in signal transduction and enhancing stress resistance, B can form a complex with sorbitol to enhance its transport capacity. This study found that, under high-temperature stress, the content of various mineral elements, including Ca and B, in fruits significantly decreased. Therefore, one of the reasons why high temperatures affect the occurrence of watercore is that they inhibit the accumulation of mineral elements. In addition, interestingly, we also found that the content of Fe was significantly increased. In plants, abiotic stress led to excessive accumulation of ROS in cells and triggered ferroptosis-like cell death. The ferroptosis-like cell death was to increase Fe accumulation and accelerate lipid membrane oxidation and cell death, so the increase in Fe content may be related to high-temperature-induced ferroptosis cell death in fruit pulp.

Sorbitol transporter (SOT) and sorbitol dehydrogenase (SDH) are responsible for the transmembrane transport and cytoplasmic degradation of sorbitol, respectively. In the current study, all SOT and SDH genes were significantly down-regulated. As mentioned above, although high temperatures or mineral element deficiencies can cause watercore, the direct cause of watercore is related to the excessive accumulation of sorbitol in the intercellular space [30]. The sorbitol in the leaves is transported through the phloem and unloaded into the extracellular space of the fruit pulp cells and then transported into the cells through SOT proteins on the cell membrane [31,32]. The intracellular sorbitol can be degraded by SDH into monosaccharides, stored in vacuoles, or used for respiration. However, when SOT is inhibited, the transport capacity of sorbitol is hindered, and a large amount of sorbitol accumulates in the intercellular space, leading to the accumulation of water and the occurrence of watercore [33]. At present, research has found that there are many factors that affect the transport of sorbitol or the activity of SOT proteins, among which calcium deficiency and boron deficiency are considered important causes of watercore [29]. Furthermore, high temperatures can lead to an excessive accumulation of ROS, which may also suppress the activity of cell membranes and membrane proteins, including SOT. The waterlogging of fruits itself is not harmful, but, due to the varying internal tissue structures of fleshy fruits such as pears and apples, oxygen diffusion resistance can occur, resulting in a significant oxygen gradient within the flesh tissue. Particularly in pear fruits, the porosity is much lower than in other Rosaceae fruits [34]. Our research group previously found that the occurrence of watercore can exacerbate the formation of an oxygen gradient, causing insufficient exchange of carbon dioxide and oxygen, resulting in physiological hypoxia stress on fruit flesh tissue [35]. Consequently, this can lead to disruptions in fruit respiratory metabolism, abnormal energy metabolism, excessive accumulation of bitter amino acids, aldehydes, and reactive oxygen species and further induce ROS generation, suppressing the expression of SOT in surrounding healthy fruit flesh and accelerating the expansion of the water-stained area. Therefore, reducing the accumulation of ROS caused by high-temperature stress in ripening fruits and enhancing the transport capacity of sorbitol are key strategies for reducing the incidence of watercore.

Heat shock transcription factors (HSFs) and heat shock proteins (HSPs) play a crucial role in regulating plant heat tolerance. HSP is a highly conserved group of proteins. They act as molecular chaperones. Under normal circumstances, they contribute to protein folding, assembly, and degradation, maintaining the stability of the proteome. During heat stress, an increase in HSP levels helps protect other proteins from denaturation and aggregation. They refold misfolded proteins to prevent the formation of non-functional protein aggregates, ensuring the normal functioning of cellular processes and maintaining plant physiological functions [36]. HSFs can interact with HSPs through protein interactions or transcriptional regulation, jointly regulating plants’ ability to adapt to high temperatures [37]. This study identified multiple PpHSFs and PpHSPs that respond to high-temperature stress, especially the identified PpHSPs that are up-regulated, and eight PpHSPs have expression levels of more than five-fold. These heat shock proteins may play a key role in resisting high-temperature stress.

## 4. Materials and Methods

### 4.1. Plant Materials and Treatment

In total, 105 days after flowering, select 20 fruits from three ‘Housui’ pear trees of comparable size (weight ranging from 250 g to 300 g with fruit shape index of around 0.9) and uniform sunlight exposure for the experiment. Place a constant-temperature resistor approximately 1 cm away from each fruit in all four cardinal directions. The resistor operates at a temperature of 80 °C, mimicking extreme high-temperature conditions in the field. Maintain this process for 15 consecutive days. Upon harvesting, the sample was immediately sliced horizontally, photographed, and the fruit’s firmness was measured. Following data recording, the fruit was peeled, the flesh was diced into small pieces, flash-frozen with liquid nitrogen, and then stored at −80 °C for subsequent analysis.

### 4.2. Physiological Assay

The surface temperature of the fruit was measured using a thermal imaging thermometer (HIKVERSION Co. Ltd., Hangzhou, China). H_2_O_2_ content was determined by colorimetric method, and the absorbance value was measured at 405 nm using a spectrophotometer (Jiancheng Co. Ltd., Nanjing, China). The determination of SOD enzyme activity was carried out using the hydroxylamine method, and the change in absorbance was measured at 550 nm (Jiancheng Co. Ltd., Nanjing, China). The activity of CAT was measured using assay kits according to the manufacturer’s instructions by ammonium molybdate colorimetric method, and the change in absorbance was measured at 405 nm (Comin Co. Ltd., Suzhou, China). The MDA content and activity of POD and APX were evaluated according to the method described by Cao et al. [38]. MDA content was measured using the thiobarbituric acid (TBA) method. The absorbance values of the supernatant were measured at 450 nm, 532 nm, and 600 nm. The determination of APX was carried out by spectrophotometric method. Measure the absorbance variation in the reaction system at 290 nm. Peroxidase activity was determined using guaiacol method, and the absorbance values of the supernatant were measured to be 470 nm. Use inductively coupled plasma mass spectrometry (ICP-MS) to determine the content of potassium (K), calcium (Ca), magnesium (Mg), phosphorus (P), boron (B), iron (Fe), zinc (Zn), and other elements in the sample [39]. Each sample undergoes three technical replicates.

### 4.3. RNA Extraction and RNA Sequencing

High-temperature-treated pulp samples (HT) and normal temperature condition pulp samples (NT) were collected, and total RNA was extracted from about 1 g sample using a TRIzol reagent kit (Invitrogen, Carlsbad, CA, USA) following the manufacturer’s instructions. RNA integrity was assessed on an Agilent 2100 Bioanalyzer (Agilent Technologies, Palo Alto, CA, USA) and confirmed by RNase-free agarose gel electrophoresis. Subsequently, mRNA was enriched, fragmented, and reverse-transcribed into cDNA using the NEBNext Ultra RNA Library Prep Kit for Illumina. The resulting double-stranded cDNA fragments were endrepaired, ligated to Illumina sequencing adapters, and purified using AMPure XP Beads (1.0X). After PCR amplification, the cDNA library was sequenced on an Illumina NovaSeq 6000 platform by Gene Denovo Biotechnology Co. (Guangzhou, China). Three biological replicates of each sample (HT and NT) were used for RNA-seq analysis. The data were submitted to the Sequence Read Archive (SRA) of NCBI database under the accession ID PRJNA1192333. Expression abundance and variations were quantified using the fragment per kilobase of transcript per million mapped reads (FPKM) value, which was calculated with RSEM software (v1.3.0). Differential expression analysis of two groups was performed using the DESeq2 R package (1.20.0), and *p*-values were adjusted to control the false discovery rate. The differentially expressed genes (DEGs) were screened according to the following criteria: |log_2_fold change| ≥ 1 and corrected *p* ≤ 0.05.

### 4.4. Statistical Analysis

SPSS 20.0 was used to analyze variance (ANOVA). Comparing means was performed with *t*-test and Duncan’s multiple range test (*p* < 0.05). GraphPad7.0 was used for charting.

### 4.5. LC-MS/MS Analysis

The LC-MS/MS method was used to analyze the changes in metabolites in the pulp of sand pear under high-temperature stress. Each sample was weighed at 0.1 g and ground into fine powder with a mortar and pestle. In total, 1000 μL of methanol/acetonitrile/H_2_O (2:2:1, *v*/*v*/*v*) was added to homogenized solution for metabolite extraction. The mixture was centrifuged for 15 min (14,000× *g*, 4 °C). The supernatant was dried in a vacuum centrifuge. Three biological replicates of each sample (HT and NT) were used for LC-MS analysis. For LC-MS analysis, the samples were re-dissolved in 100 μL acetonitrile/water (1:1, *v*/*v*) solvent. Analysis was performed using a UHPLC (1290 Infinity LC, Agilent Technologies) coupled to a quadrupole time-of-flight (AB Sciex TripleTOF 6600). A variable importance in projection (VIP) score of (O)PLS model was applied to rank the metabolites that best distinguished between two groups. The threshold of VIP was set to 1. In addition, *t*-test was also used as a univariate analysis for screening differential metabolites. Those with a *p*-value of *t*-test < 0.05 and VIP ≥ 1 were considered differential metabolites between two groups.

## 5. Conclusions

This study conducted a comprehensive analysis of the mechanism by which mature pear fruits respond to high-temperature stress through multiple omics analyses. The results indicate that high temperature can lead to abnormal fruit ripening, inhibit mineral element accumulation and sorbitol metabolism, and thus trigger the formation of watercore. In addition, we identified multiple HSP and HSF genes that respond to high-temperature stress. This study provides a new perspective for investigating the mechanism of high-temperature tolerance in sand pear fruits.

## Figures and Tables

**Table 1:** Differential metabolites of sugars and organic acids in fruits under high-temperature stress.

Sugars and Organic Acid Metabolites	Metabolite	Log_2_^FC^	VIP
Sugars	Fructose	0.24	1.83
	Sucrose	3.35	33.36
	Sorbitol	0.32	32.71
Organic Acids	Malic acid	−0.62	19.88
	Shikimic acid	−0.47	2.81
	Succinic acid	1.67	8.04
	Quinic acid	−0.09	7.83
	Fumaric acid	−0.64	4.90

**Table 2:** Differential metabolites of bioactive substances in fruits under high-temperature stress.

Metabolite	Log_2_^FC^	VIP
Pipecolic acid	−1.11	3.69
γ-aminobutyric acid	−0.46	7.16
Methionine	−0.72	4.04
Alanine	−1.11	2.99
Asparagine	−3.28	14.22
Aspartic acid	−1.85	8.97
Serine	−0.44	1.19
Glutamic acid	−0.33	1.34
Isoleucine	0.311	2.023
Quercetin	−0.83	1.66
Chlorogenate	−1.10	1.06
Gallic acid	−9.03	6.86

**Table 3:** Differentially expressed antioxidant enzyme genes in pear fruits under high temperature.

Function	ID	Log_2_^FC^
Peroxidase, POD	LOC103966973	−2.58
	LOC103934252	−2.34
	LOC103964015	1.04
	LOC103938713	3.24
	LOC103958862	4.91
Superoxide dismutase, SOD	LOC103947719	−2.28
	LOC103956225	1.12
Ascorbate peroxidase, APX	LOC103934327	1.58
	LOC103948906	1.83
Glutathione S-transferase, GST	LOC103930887	1.48
	LOC103958810	8.74

**Table 4:** Differentially expressed sucrose and sorbitol metabolism genes in pear fruits under high-temperature stress.

Function	ID	Log_2_^FC^
Sucrose-phosphate synthase, SPS	LOC103952486	2.08
Sucrose synthase, SS	LOC103935319	1.27
	LOC103959924	−1.73
	LOC103939687	−3.60
Neutral invertase, NI	LOC103952542	−1.45
	LOC103954772	−1.06
	LOC103928220	1.38
Sorbitol dehydrogenase, SDH	LOC103933317	−1.05
	LOC103933318	−3.64
	LOC103933319	−2.05
	LOC103960508	−2.31
	LOC103960512	−2.25
	LOC103960507	−2.05
	LOC103960513	−1.69
	LOC103933316	−3.14
	LOC103933312	−2.58
	LOC103933314	−3.70
Sorbitol transporter, SOT	LOC103929477	−3.41
	LOC103964608	−1.23
	LOC103934620	−1.38
	LOC103936910	−1.49

**Table 5:** Differentially expressed ethylene and ABA biosynthesis genes in pear fruits under high-temperature stress.

Function	ID	Log_2_^FC^
1-Aminocyclopropane-1-carboxylate Oxidase, ACO	LOC103946002	2.42
	LOC103958907	3.64
	LOC103939367	2.96
	LOC103954990	−1.62
	LOC103943975	14.23
	LOC103967481	1.20
	LOC103958927	2.13
	LOC103958906	2.71
	LOC103943060	1.23
Zeaxanthin Epoxidase, ZEP	LOC103937745	−1.50
9-cis-Epoxycarotenoid Dioxygenase, NCED	LOC103945979	−2.07
Abscisic Aldehyde Oxidase, AAO	LOC103959275	2.40
	LOC103952077	−4.43

**Table 6:** Differentially expressed heat shock proteins (HSPs) and heat shock transcription factors (HSFs) in pear fruits under high-temperature stress.

Function	ID	Log_2_^FC^
Heat shock protein, HSPs	LOC103927502	2.50
	LOC103927503	2.81
	LOC103927504	3.71
	LOC103936175	1.78
	LOC103936185	3.11
	LOC103936191	2.94
	LOC103936226	4.62
	LOC103942700	1.70
	LOC103945002	1.26
	LOC103945384	2.51
	LOC103948411	2.46
	LOC103952522	3.24
	LOC103955091	1.51
	LOC103956001	4.71
	LOC103956628	1.86
	LOC103958215	2.11
	LOC103963260	2.11
	LOC103964327	5.40
	LOC103966141	5.32
	LOC103936199	3.66
	LOC103940225	3.04
	LOC103946415	5.03
	LOC103955267	2.89
	LOC103942709	2.67
	LOC103942692	1.31
	LOC103945383	3.70
	LOC103927498	5.73
	LOC103953301	9.52
	LOC103960954	3.45
	LOC103949874	11.39
	LOC103960307	11.04
	LOC103961953	3.50
	LOC103966169	5.84
	LOC103959824	3.00
Heat shock transcription factors, HSFs	LOC103944300	4.22
	LOC103952243	2.67
	LOC103955174	2.49
	LOC103944219	2.24
	LOC103960090	1.67
	LOC103936188	−1.12
	LOC103945943	−1.19

## Data Availability

The data presented in this study are available upon request from the corresponding author.
